# Early and late manifestations of neuropathy due to *HSPB1* mutation in the Jewish Iranian population

**DOI:** 10.1002/acn3.51362

**Published:** 2021-05-11

**Authors:** Lior Greenbaum, Merav Ben‐David, Vera Nikitin, Orna Gera, Ortal Barel, Adi Hersalis‐Eldar, Jana Shamash, Noam Shimshoviz, Haike Reznik‐Wolf, Mordechai Shohat, Dan Dominissini, Elon Pras, Amir Dori

**Affiliations:** ^1^ The Danek Gertner Institute of Human Genetics Sheba Medical Center Tel Hashomer Israel; ^2^ The Joseph Sagol Neuroscience Center Sheba Medical Center Tel Hashomer Israel; ^3^ Sackler Faculty of Medicine Tel Aviv University Tel Aviv Israel; ^4^ Department of Neurology Sheba Medical Center Tel Hashomer Israel; ^5^ The Genomic Unit Sheba Cancer Research Center, Sheba Medical Center Tel Hashomer Israel; ^6^ Wohl Institute of Translational Medicine Sheba Medical Center Tel Hashomer Israel

## Abstract

**Objective:**

Mutations in the *HSPB1* gene are associated with a distal hereditary motor neuropathy type 2 (dHMN2) or Charcot‐Marie‐Tooth disease type 2F (CMT2F), usually with autosomal dominant inheritance. This study aimed to describe the phenotype of the *HSPB1* c.407G>T (p.Arg136Leu) mutation at early and late stages of the disease course.

**Methods:**

We identified this mutation (previously reported in patients from Italy) in a heterozygous state, among 14 individuals from eight families of Jewish Iranian descent. The clinical, electrophysiological and ultrasonographic features were evaluated during early (less than 5 years, N = 9) or late disease course (N = 5).

**Results:**

The majority of subjects were males with a mean age at onset of 43.4 years (range 21‐67). Common initial symptoms were gait imbalance, distal (often asymmetric) lower limb weakness and feet numbness. Neurological examination in early disease course showed distal lower extremity weakness in nearly all cases, and absent Achilles tendon reflex in about half. A minority had distal loss of pain, vibration or position sensation. These findings were more prevalent in late disease stage. Electrodiagnostic studies demonstrated a length‐dependent axonal motor neuropathy, with typical preferential involvement of the tibial nerve. Muscle ultrasound showed a corresponding length‐dependent increase of homogeneous echo‐intensity, most noticeably in the gastrocnemius. One patient had a dual diagnosis of CMT2F and CMT2W.

**Interpretation:**

The *HSPB1* c.407G>G (p.Arg136Leu) mutation causes an adult‐onset, predominantly motor, axonal neuropathy in individuals of Jewish Iranian descent. Variable manifestations are noticed, and sensory involvement is more prominent in prolonged disease duration.

## Introduction

Mutations in the heat‐shock protein beta 1 (*HSPB1*) gene, also known as *HSP27*, are associated with the overlapping conditions of distal hereditary motor neuropathy type 2 (dHMN2) and Charcot‐Marie‐Tooth disease type 2F (CMT2F).^1^, ^2^, ^3^, ^4^ Both phenotypes are characterized by a slowly progressing, distal, muscle weakness and wasting (predominantly lower limb), but differ by the presence of sensory involvement in the latter. Electrophysiological studies show a length‐dependent axonal motor or motor‐sensory neuropathy, respectively.^2^, ^5^ Since its initial description,^6^ multiple pathogenic mutations throughout *HSPB1* (chromosome 7q11.23) have been reported worldwide, usually of the missense type, with autosomal dominant and rarely with autosomal recessive inheritance.^6^, ^7^, ^8^ Age of onset was variable, and heterogenous involvement of sensory symptoms and other neurological features were described, without a clear genotype‐phenotype correlation.^2^, ^3^, ^9^, ^10^

The c.407G>T (p. Arg136Leu) mutation, located in exon 2 of *HSPB1*, was previously reported in heterozygous state among patients from Italy.^11^, ^12^, ^13^, ^14^ It results in an arginine‐to‐leucine substitution in the α‐crystalline domain of the protein. In general, affected individuals had late‐onset gait difficulties, distal muscle atrophy and weakness of the lower limbs, sensory disturbances and occasionally deafness and pyramidal signs.^11^, ^12^, ^13^, ^14^

We identified the *HSPB1* c.407G>T (p. Arg136Leu) mutation in 14 individuals from eight families of Jewish Iranian descent. The mutation has not been previously reported in this population. We describe the clinical manifestations in our sample, characterized by progressive distal weakness, but also with variable sensory involvement or other features. The study highlights the initial complaints and findings that appeared during the first 5 years of the disease course, and emphasize electrodiagnostic and ultrasound characteristics.

## Methods

### Participants

In this retrospective cross‐sectional study, we enrolled all known heterozygous individuals with the NM_001540.5*:HSPB1* c.407G>T (p. Arg136Leu) mutation identified at the genetic institute and/or the neuromuscular clinic at Sheba Medical Center (SMC), Tel Hashomer, Israel. This is a tertiary referral hospital for patients with neuromuscular disorders. The study was approved by the institutional review board.

Molecular diagnosis was performed as a clinical service, following genetic counselling. In four families, the mutation was identified by whole‐exome sequencing (WES) to the index case, followed by segregation analysis for other relevant family members. In the other four families, the mutation was suspected and therefore tested by targeted Sanger sequencing. All subjects provided an informed consent.

Detailed medical history was obtained for all families. Subjects were examined by a board‐certified neurologist, and CMT examination score^15^ was calculated. Nine cases were evaluated during the first 5 years of disease course, defined by us as early stage. The other five were evaluated in the later stage of disease (>5 years from onset).

The c.407G>T (p. Arg136Leu) mutation frequency in the Jewish Iranian population living in Israel was determined by testing 150 unrelated controls from this origin, who performed prenatal carrier screening at the SMC genetic institute. Their medical history was unavailable.

### Electrophysiological studies

Nerve conduction studies (NCSs) and Electromyography (EMG) for 12 of the mutation carriers were performed at the SMC neuromuscular clinic. Studies employed Natus EMG equipment (Viking EDX, Madison, USA), measuring compound muscle action potential (CMAP) and antidromic sensory nerve action potential (SNAP) following stimulation of motor and sensory nerves, respectively, according to established guidelines. For all studies, limb temperature was above 32°C. Frequency filters were set to 3Hz (low) and 10 kHz (high) for motor studies and 2 Hz (low) and 2 kHz (high) for sensory studies. Normal values were based on published^16^ and local lab data. CMAP and SNAP amplitudes were both measured from onset‐to‐peak, whereas latencies were determined by onset and peak, respectively. Sensory polyneuropathy was diagnosed when bilateral sural or unilateral sural and superficial peroneal SNAPs were below 5 μV, with sural/radial amplitude ratio below 0.2.

### Nerve and muscle ultrasound studies

High‐resolution ultrasound was performed using a linear transducer (frequency band 5–18 MHz; Phillips Affiniti 70). The cross‐sectional area (CSA) of the median and ulnar nerves was measured at the wrist, mid‐forearm, and mid‐arm. The ulnar was also assessed at the elbow. Normal values were based on published values,^17^ modified according to our local lab parameters. Muscle evaluation included the medial gastrocnemius, tibialis anterior, vastus lateralis, biceps brachii and abductor digiti minimi. Nerves and muscles were examined on one side.

Estimation of muscle degeneration due to denervation was assessed by quantification of muscle echo‐intensity and homogeneity with the National Institute of Health (NIH) image software histogram analysis (https://imagej.nih.gov/ij/), expressed as means and standard deviations. Increased echo‐intensity represents the replacement of muscle fibers by connective tissue. This initially occurs in a non‐homogenous pattern throughout the muscle, presumably due to regional atrophy and hypertrophy, secondary to ongoing denervation and reinnervation, respectively.^18^ Further muscle degeneration results in a more homogeneous appearance, though not necessarily with an additional increase in echo‐intensity. To take into account this chronic homogenous change in muscle architecture, the mean echo‐intensity value was divided by the standard deviation, resulting in an intensity factor.

## Results

### Clinical description

Fourteen heterozygous NM_001540.5:c.407G>T (p. Arg136Leu) mutation carriers from eight families were identified. Detailed demographic and clinical data for each are provided in Table 1. Most subjects were male (9/14; 64.3%). The mean age of symptoms onset was 43.4 ± 13.8 years (range 21–67), and mean disease duration at clinical evaluation was 6.6 ± 6.1 years (range 0–17). Two of the mutation carriers had no persistent complaints but did show subtle findings on clinical examination.

**Table 1:** Demographic and clinical data. All cases are heterozygous for the *HSPB1* c.407G>T (p. Arg136Leu) mutation.

Family	Case	Sex	Age	Age of onset	Dur (y)	Other disorders	Initial symptom	Muscle atrophy	Side	TA (MRC)	Ankle DTR	Pain sense	Vib sense	Prop sense	Toe walk	Ankles walk	CMT exam score
A	II1	F	57	40	17	T2D	Ankle weakness, sprained ankles, limp, falls	TA, FDI, GC	R	0	0	socks	Low	Absent	WC		20
									L	0	0		Low	Absent			
A	II2	M	54	48	2	T2D, obesity	Pain in feet followed by gait imbalance	None	R	3.5	0	NL	NL	NL	Can't	Can't	4
									L	3	0	NL	NL	NL	Can't	Can't	
A	II3	F	53	52	1		Pain in feet when walking	None	R	5	1	NL	NL	NL	Impaired	NL	1
									L	5	1	NL	NL	NL	Impaired	NL	
A	II4	M	43	38	5	T2D, hearing	Muscle cramps and fatigue, numb feet, gait imbalance	feet and calfs	R	1	0	socks	Absent	Low	Can't	Can't	11
						impairment			L	1	0		Low	Low	Can't	Can't	
B	I1	F	63	63	0		Asymptomatic, intermittent numb in toe	None	R	4	2	NL	NL	NL	NL	Difficulty	2
									L	4.5	2	NL	NL	NL	NL	Difficulty	
B	II1	M	38	21	17		L ankle weakness, limp	TA, GC	R	3.5	0	socks	Low	Low	Can't	Can't	9
									L	3	0		Low	Absent	Can't	Can't	
B	II2	M	31	31	0		Asymptomatic	Hallux valgus	R	4.5	2	NL	NL	NL			0
									L	4.5	2	NL	NL	NL			
C	I1	F	53.5	48	5		R LE weakness, limp	Feet muscles	R	5	0	NL	NL	NL	NL	Impaired	2
									L	5	0	NL	NL	NL	NL	Impaired	
C	II1	F	29	26	3		Left foot weakness, bilateral pes cavus, numb/ting hands and feet	None	R	5	2	NL	NL	NL	NL	NL	2
									L	5	2	NL	NL	NL	NL	NL	
D	I1	M	76	67	9		Ankle weakness	None	R	2	0	NL	NL	NL	Can't	Can't	5
									L	3	0	NL	NL	NL	Can't	Can't	
E	I1	M	67	56	11	prediabetes, obesity	Numb feet, gait imbalance	None	R	0	0	socks, gloves	Absent	Absent	Can't	Can't	12
									L	0	0		Absent	Absent	Can't	Can't	
F	I1	M	65	30	15	CKemia	Sprained ankles, muscle stiffness and cramps	EDB	R	4.5	0	NL	NL	NL	NL	Impaired	1
									L	4.5	0	NL	NL	NL	NL	Impaired	
G	I1	M	50	49.5	3		LE weakness, numb left toe, gait imparment, falls	FDI, APB, TA, GC	R	4.5	0	NL	Low		Impaired	Impaired	3
									L	4.5	0				Impaired	Impaired	
H	I4	M	42	38	4	prediabetes, obesity	Fasciculation, Feet and ankle pain, numb feet	winging scapula	R	3.5	0	NL	Low	NL	Can't	Can't	6
									L	3	0	NL	Low	NL	Can't	Can't	

Family A includes 4 siblings, Family B a mother and two sons (Patient B‐II2 is also a carrier of the *HARS* c.464T>G (p. Val155Gly) mutation) and family C a mother and a daughter.

Blank spaces means missing data.

Abbreviations: APB, abductor pollicis brevis; CK, hyper‐CKemia; CMT, Charcot Marie Tooth; dis, disorder; DTR, deep tendon reflex; Dur, Duration; EDB, extensor digitorum brevis; FDI, first dorsal interosseous; GC, gastrocnemius; L, left; LE, lower extremity; MRC, medical research council grade; NL‐ normal; Numb‐numbness; Prop, proprioception; R, right; T2D, Type 2 diabetes; TA, tibialis anterior; Vib, vibration; WC, wheel‐chair bound.

Initial symptoms in most cases were gait imbalance (7/14) and distal lower extremity weakness (6/14), mainly affecting the ankles. Feet numbness was also a common initial symptom (6/14). Type 2 diabetes (T2D) or a prediabetes state were present in only three of these six patients, and no alternative explanation for sensory loss was identified in the remainder. Feet pain in early disease, predominantly during or after walking, was reported by 3/14 cases. Recurrent ankle sprains, falls or cramps were each reported by two participants (2/14). Asymmetry of initial symptoms was described by half of the cases. Autonomic symptoms were denied.

Details of the main neurological findings on examination are provided in Table 2, according to disease duration. Distal muscle weakness, predominantly of the tibialis anterior or extensor hallucis longus, was the most common finding, eventually present in nearly all cases (13/14), and presented during the first 5 years in the majority (8/9), including two asymptomatic carriers. Upper motor neuron signs, which raised concern of a diagnosis of amyotrophic lateral sclerosis (ALS), were seen in 3/14 patients. All but one patient (13/14) were ambulatory at the last clinical evaluation. Cognitive and cranial nerve exams were normal in all but one patient which showed hearing impairment. Additional notable findings were asymmetric scapular winging and hyper‐CKemia (up to 1200 u/l) each in a different patient.

**Table 2:** Details of main neurological findings upon examination.

Finding	During first 5 years (N = 9, 2.6 ± 1.9 years)	After 5 years of disease (N = 5, 13.8 ± 3.6 years)	All (N = 14, 6.6 ± 6.1 years)
Distal muscle atrophy	4	4	8
Fasciculation	3	2	5
Prominent pes cavus deformity	0	1	1
Distal muscle weakness (predominantly of the tibialis anterior or extensor hallucis longus)	8	5	13
Absent Achilles deep tendon reflex	5	5	10
Upper motor neuron signs*	1	2	3
Distal loss of pain sensation	1	3	4
Distal loss of vibration or position sensation	3	3	6
Abnormal Romberg sign**	3	1	4
Impaired toe or ankle walking ability	7	5	12
Impaired tandem walking	3	5	8

* Brisk deep tendon reflexes in the upper extremities or knees, extensor plantar response, positive Hoffman and Tromner signs.

** Refers to 13 ambulatory cases.

### Electrodiagnostic findings

**Figure 1:**
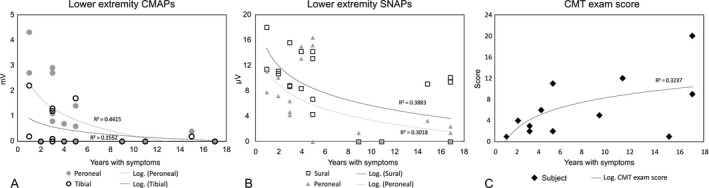
Lower extremity motor (A) and sensory (B) responses, and CMT exam score (C) according to symptom duration. Tibial versus peroneal CMAPs are commonly low or show no response, even in early disease course. Sural SNAPs are commonly preserved. The CMT exam score gradually increases with symptom duration. A logarithmic regression line is shown. Absent responses in both extremities of an individual are superimposed and marked by a single point on the zero line of the graph. CMT, Charcot‐Marie‐Tooth; CMAP, compound muscle action potential; SNAP, sensory nerve action potential.

All 12 participants who performed electrodiagnostic evaluation showed evidence of an axonal motor polyneuropathy, in some with additional sensory involvement (Table 3 and Figure 1). Evaluation was performed for seven cases in the early stage (mean disease duration: 3.3 ± 1.5), and for five in the late stage (mean disease duration: 13.8 ± 3.6).

**Table 3:**
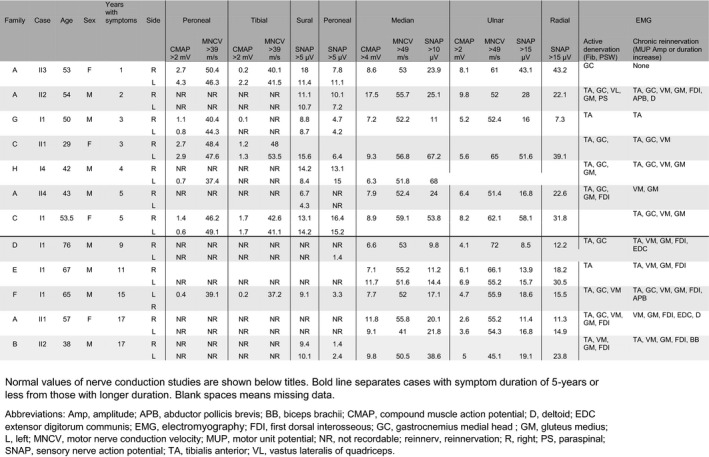
Electrophysiological features and symptom duration.

Normal values of nerve conduction studies are shown below titles. Bold line separates cases with symptom duration of 5‐years or less from those with longer duration. Blank spaces means missing data.

Abbreviations: Amp, amplitude; APB, abductor pollicis brevis; BB, biceps brachii; CMAP, compound muscle action potential; D, deltoid; EDC extensor digitorum communis; EMG, *electromyography*; FDI, first dorsal interosseous; GC, gastrocnemius medial head; GM, gluteus medius; L, left; MNCV, motor nerve conduction velocity; MUP, motor unit potential; NR, not recordable; PS, paraspinal; R, right; reinnerv, reinnervation; SNAP, sensory nerve action potential; TA, tibialis anterior; VL, vastus lateralis of quadriceps.

In the first 5 years of clinical symptoms, motor NCSs showed a small tibial CMAP in all cases, which was smaller than the peroneal CMAP in seven of nine tested extremities (4/7 patients) (Figure 1A). In four extremities (of 3 cases) both tibial and peroneal response was not detected. These responses were not detected in seven extremities of four patients with a longer disease course, and in one patient they were below 0.5 mV, with a smaller tibial CMAP amplitude.

Lower extremity SNAPs were preserved throughout the course of the disease in most cases, with normal sural response detected in 15 of 21 tested extremities. In total, sensory polyneuropathy was diagnosed in 4/12 cases (Figure 1B). In the first 5 years of symptoms, sural SNAPs were abnormal in only 1 of 13 tested extremities, but in patients with a longer disease course, this increased to five of eight extremities. Therefore, sensory polyneuropathy was diagnosed in only 1/7 of patients during the first 5 years (in a patient that had T2D). Later in the disease course, sensory polyneuropathy was diagnosed in three of five patients, of which two had prediabetes or T2D. Upper extremity CMAPs and SNAPs were essentially preserved, though ulnar CMAPs and SNAPs did show a gradual decline.

In all but one case, needle EMG showed signs of ongoing/active denervation in the lower limb with a length‐dependent pattern, commonly on the background of normal SNAPs. Active or chronic denervation/reinnervation in the first dorsal interosseous were identified in only two cases with a disease duration of less than 5 years at the time of EMG, but in all cases with a longer disease course (5/5). None of these patients showed active denervation in proximal muscles of the arm (biceps brachii or deltoid). Fasciculation was seen in two patients. Paraspinal muscles were tested in four patients, and fibrillation potentials were identified in only one.

### Ultrasound examination

Six cases were evaluated by ultrasound: three in early disease stage (mean disease duration: 2.3 ± 1.5) and three in the late stage (mean disease duration: 13.7 ± 4.2).

A mild thickening of nerves was noted at entrapment sites in three cases. These three had an increase in CSA of the ulnar nerve at the elbow (up to 26% of the upper limit of normal). Two showed a mild increase in CSA of the median nerve at the wrist as well as the arm (up to 17% and 15%, respectively).

**Figure 2:**
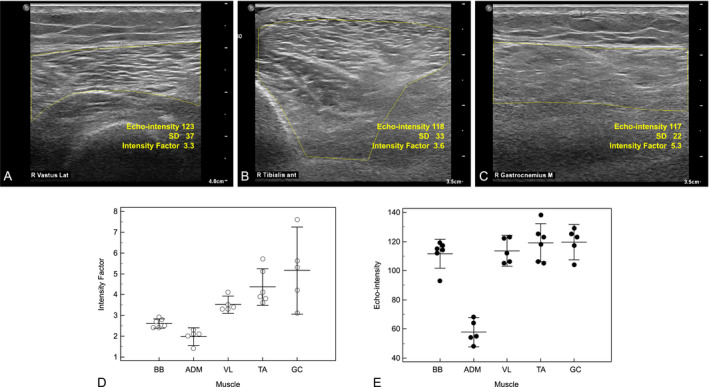
Muscle Ultrasound images of the quadriceps vastus lateralis (A), tibialis anterior (B) and medial gastrocnemius (C). Echo‐intensity value, homogeneity depicted by standard deviation and the calculated intensity factor are shown. The gastrocnemius shows loss of muscle architecture, with echo‐intensity that is similar to that of the tibialis anterior and vastus medialis. The gastrocnemius intensity factor is increased in comparison to these muscles. The intensity factor (D) and echo‐intensity (E) of proximal and distal muscles of the upper and lower limbs. A length‐dependent pattern for the lower limb is shown by calculation of the intensity factor. ADM, abductor digiti minimi; ant, anterior; BB, biceps brachii; GC, gastrocnemius medial head; Lat, lateralis; M, medial; SD, standard deviation; TA, tibialis anterior; VL, vastus lateralis of quadriceps.

Muscle echo‐intensity evaluation showed increased homogeneous echo‐intensity, defined here by an intensity factor (Figure 2A‐C), with a length‐dependent pattern in all tested subjects (Figure 2D). This was not apparent by quantifying “raw” echo‐intensity alone (Figure 2E). Since symptom duration at ultrasound examination was less than 5 years for three cases, this finding may be a sensitive marker for distal denervation. Interestingly, the intensity factor of the medial gastrocnemius was substantially higher in comparison to that of the tibialis anterior (Figure 2C vs. B) in all but patient B‐II2 (discussed next), consistent with the typical, more prominent involvement of tibial versus peroneal nerve. In three patients, muscle ultrasound showed frequent fasciculation, mainly in the quadriceps.

### A patient with dual CMT diagnosis

Patient B‐II1 inherited the *HSPB1* mutation from his mother. In addition, he had a heterozygous mutation, NM_002109.6:c.464T>G (p. Val155Gly) in the *HARS* gene. This mutation, causing CMT type 2W (CMT2W), was paternally inherited, indicating a dual diagnosis of CMT2F and CMT2W. The c.464T>G (p. Val155Gly) mutation in *HARS1* was previously reported in a family of Jewish Iranian descent.^19^

Both parents of the index case exhibited a mild motor neuropathy. The mother (B‐I1) was interviewed at age 63 and reported only intermittent sensory symptoms but exhibited mild distal weakness, absent ankle reflexes and difficulty with ankle walking upon examination. In contrast, her son with both *HSPB1* and *HARS1* heterozygous mutations presented with severe early onset (age 21) phenotype of asymmetric distal lower extremity weakness. He had no evidence of T2D or other acquired causes of neuropathy. The weakness gradually worsened, and at the age of 33 he had asymmetric bilateral foot drop, prominent distal lower and mild upper limbs weakness and fasciculation, distal sensory loss (reduced pain, vibration and position sense) and upper motor neuron signs, including positive extensor plantar response. Electrodiagnostic studies performed 17 years after the onset of symptoms showed no tibial or peroneal response, while sural SNAPs were normal. EMG demonstrated fasciculation in multiple muscles (tibialis anterior, vastus medialis, gluteus medius, and first dorsal interosseous), as well as chronic motor unit changes, predominantly in distal muscles. Ultrasound of muscles showed an increased intensity factor with a length‐dependent pattern, which was most prominent in the tibialis anterior.

This *HARS1* mutation was excluded in all other 13 participants by WES or Sanger sequencing.

### Haplotype analysis and mutation frequency

Haplotype analysis for three of the families with available WES data revealed a shared haplotype of at least 7.85 Mb that included the *HSPB1* gene (minimal size position: 75,554,104‐83,408,435, according to GRCh38). The haplotype was constructed using 20 single nucleotide variants, all located in exons. This supports a common founder for these families.

We screened 150 unrelated controls of Jewish Iranian descent for the *HSPB1* c.407G>T (p. Arg136Leu) mutation, but it was not detected in any of them. Furthermore, the mutation was absent in additional 38 Jewish Iranian samples included in our in‐house exome database. The overall frequency of this variant in gnomAD database (https://gnomad.broadinstitute.org/) was 0.0004%.

## Discussion

We describe here the clinical, electrophysiological, and ultrasonographic features of 14 individuals of Jewish Iranian descent, with the *HSPB1* c.407G>T (p. Arg136Leu) mutation, causing a dominantly inherited neuropathy.

Common initial symptoms were related to distal weakness, leading to gait imbalance in nearly half of the cases, and feet numbness. Examination in the early disease course revealed distal lower limbs muscle weakness and mild gait dysfunction, but sensory abnormalities were found in a minority of patients. However, with the progression of the disease, sensory findings became more common. Similarly, the hallmark of NCSs in the early years was a distal reduction of motor responses, whereas sensory responses were preserved. In later years, sensory responses were lost in some of the patients, which could not be attributed to other medical conditions. The sensory symptoms and findings upon neurological examination and electrophysiology indicate that in nearly half of the patients, this specific *HSPB1* mutation caused a length‐dependent motor and sensory polyneuropathy, with motor predominance, consistent with CMT2F. In those patients that remain with no sensory symptoms and findings, dHMN2 may be more correctly diagnosed. The continuum between the two conditions is recognized.^1^, ^2^, ^4^

Preferential involvement of tibial over peroneal CMAPs was previously reported in one patient^2^ but not systematically addressed. We found this feature early in the course of the disease, while later on, a response from both nerves could not be elicited. This predominance was not clinically apparent, as plantar flexion was weaker than dorsiflexion in only six extremities. Therefore, this finding may serve as an important diagnostic clue, potentially specific to this type of neuropathy.^9^

In addition, ultrasound imaging showed preferential degeneration of the medial gastrocnemius versus tibialis anterior muscle. While this was visually apparent, raw densitometry analysis alone could not detect this difference, as the confluent changes in muscle due to widespread denervation‐degeneration were not necessarily associated with a further increase in echo‐intensity. It was taken into account by calculating the variability of echo‐intensity, allowing identification of a length‐dependent pattern and gastrocnemius over tibialis anterior structural muscle changes. This is consistent with previously reported magnetic resonance imaging (MRI) findings, which showed severe fatty replacement of posterior compartment muscles – medial gastrocnemius and soleus, while the anterior compartment was not as severely involved.^9^, ^13^, ^20^ Interestingly, Gaeta et al.^13^ noted an asymmetry in some of the patients, in agreement with the clinical description in our sample.

The *HSPB1* c.407G>T (p. Arg136Leu) mutation was previously described in Italian patients,^11^, ^12^, ^13^, ^14^ which had symptoms that were generally similar to those of our patients. Nevertheless, this mutation has not yet been reported in the Jewish Iranian population, to the best of our knowledge. Identification of 14 mutation carriers in a single‐center offered a valuable opportunity for a comprehensive and refined phenotyping. However, longitudinal data about disease progression in our subjects was unavailable, and future studies in this direction are required.

We found a great variability among the participants, even within the same family, including for age of onset, weakness severity, presence of sensory signs and symptoms and additional features (fasciculation, pes cavus, hearing impairment, hyperreflexia and hyper‐CKemia). In a single patient, the pronounced intrafamilial variability was attributed to a dual diagnosis of CMT2F and CMT2W. He presented with an early and severe phenotype, while his mother, who had the *HSPB1* mutation, considered herself asymptomatic at the age of 63, and exhibited only mild distal lower extremity muscle weakness.

Fasciculation have been reported and/or identified in more than a third of our cases. The combination of muscle weakness with fasciculation, prominent active denervation on EMG, preserved sensory responses and upper motor neuron signs (such as hyperreflexia) could lead to the mistaken diagnosis of ALS. In these cases, a relevant family history may be pivotal to the correct diagnosis, particularly in Jews from Iranian descent. Moreover, *GNE* myopathy, which is also common in this population, needs to be kept in mind,^21^, ^22^ and was initially suggested in one of our cases. Upon electrodiagnostic testing, neurogenic features rule out myopathy and preferential involvement of the tibial over peroneal distribution (as discussed above), and absence of active denervation in proximal upper limbs, aid in the differentiation from ALS. Furthermore, slow progression and sensory symptoms should raise suspicion of CMT2F.

Interestingly, a male predominance for CMT2F/dHMN2 was suggested, in terms of disease severity and penetrance, even for this specific mutation.^11^, ^13^ Most of our cases were males, but we did not systematically screen non‐affected family members for the mutation, and therefore cannot compare the frequency of asymptomatic male and female carriers. Notably, the only non‐ambulatory patient was a female (with age at onset of 40 years), and in another female patient the age at onset was 27 years.

The protein encoded by the *HSPB1* gene, Hsp27, is a member of the small heat‐shock proteins family, ubiquitously expressed in various tissues, including neurons.^1^, ^23^, ^24^ It is a chaperon that binds to misfolded or denatured proteins and prevents their aggregation.^23^ The disease pathology is related to altered chaperon function, and involves neurofilament dysregulation and dysfunction of microtubules and mitochondria.^3^, ^23^, ^24^, ^25^

Updated lists of CMT2F/dHMN2 causing mutations in *HSPB1* are provided elsewhere.^1^, ^2^, ^3^, ^25^ Many of them are located in proximity to our mutation, involving amino acids 127‐129, 135 and 140‐141. Another mutation in amino acid 136 was described by Evgrafor et al., leading to the substitution of Arginine to Tryptophan in a Belgian family.^6^ This region is positioned in the α‐crystalline domain, and mutations within it may affect the chaperon activity, monomerization and affinity to other proteins.^3^, ^25^

To conclude, our findings show that the *HSPB1* 407G>T (p. Arg136Leu) mutation is a cause of distal, predominantly motor, axonal neuropathy with late onset among individuals of Jewish Iranian descent. This diagnosis should be therefore considered in the relevant circumstances.

## Conflicts of Interest

The authors do not have any conflict of interest.
